# Emotional Intelligence and the Big Five as Predictors of Students’ Performance in Collaborative Problem Solving

**DOI:** 10.3390/jintelligence13090109

**Published:** 2025-08-29

**Authors:** Ana Altaras, Zorana Jolić Marjanović, Kristina Mojović Zdravković, Ksenija Krstić, Tijana Nikitović

**Affiliations:** Department of Psychology, Faculty of Philosophy, University of Belgrade, 11000 Belgrade, Serbia; zjolic@f.bg.ac.rs (Z.J.M.); kristina.mojovic@f.bg.ac.rs (K.M.Z.); kkrstic@f.bg.ac.rs (K.K.); tijana.nikitovic@f.bg.ac.rs (T.N.)

**Keywords:** emotional intelligence, personality, Big Five, collaborative problem solving, secondary-school students

## Abstract

We examined the effects of emotional intelligence (EI) and the Big Five on students’ performance in collaborative problem solving (CPS). 162 secondary-school students completed the Mayer-Salovey-Caruso Emotional Intelligence Test and the Big Five Inventory. Divided into 54 triads (64.8% female), they then collaboratively solved a complex social problem. Based on video-recordings of the CPS sessions, we assessed four CPS processes: the team’s socio-cognitive exchange, socio-emotional interaction, task management, and relationship management. The CPS product (solution) of each team was judged by two independent raters. Using structural equation modelling (1) with team-level EI abilities as predictors, we found a small indirect effect (via CPS processes) of both understanding and managing emotions on the CPS product, and a medium-size direct effect of understanding emotions on the same criterion; (2) with team-level traits as predictors, a medium-size positive effect of neuroticism on task management, a small negative effect of extraversion on relationship management, and a small positive effect of openness on the CPS product. A model including both EI and personality confirmed their independent contributions to CPS performance, with EI abilities contributing both directly and indirectly to the CPS product, and the contribution of personality narrowed down to neuroticism positively affecting task management.

## 1. Introduction

Academic performance is a complex outcome variable, the prediction of which has been a long-standing challenge in psychology. Needless to say, intelligence holds its place as the “number one” (both earliest established and most important) dispositional predictor of this outcome. Indeed, this is why the abilities captured with traditional IQ tests (i.e., the abilities to process verbal, numerical/quantitative, and figural-spatial information) are more specifically referred to as “academic intelligence”.

### 1.1. Beyond Academic Intelligence: Emotional Intelligence and Personality as Predictors of Academic Performance

However, after nearly a century of focusing almost exclusively on academic intelligence, attention has shifted to another set of cognitive abilities—namely, those geared for processing emotion-related information and dubbed “emotional intelligence” (EI). According to the Mayer-Salovey model (e.g., Mayer et al. 2016), the abilities constituting EI can be organized into four “branches”, representing four problem-solving areas. These are as follows: (1) perceiving emotions—picking up and decoding verbal/nonverbal emotional cues, and vice versa, encoding emotions into appropriate expressions; (2) using emotions—aligning emotions with cognitive processes (e.g., sensations, judgments, decision making), thus using them as a vehicle for thought and action; (3) understanding emotions—knowledge of emotion words and emotion patterns and reasoning based on this knowledge; (4) managing emotions—knowledge of how emotions can be effectively regulated to achieve the desired goal.

Apart from supporting the status of EI as another broad (second-order) factor within the structure of cognitive abilities (Evans et al. 2020; MacCann et al. 2014), empirical research has validated it as a positive predictor of social adaptation/functioning (Brackett et al. 2006; Lopes et al. 2004) and psychological well-being (e.g., Altaras Dimitrijević et al. 2018b). But why would a “non-academic” intelligence matter in predicting academic performance? Several paths have been proposed by which EI may influence this outcome as well (MacCann et al. 2020; see also Ivcevic and Brackett 2014; Lopes et al. 2012; Mestre et al. 2006). First, EI is likely to be involved in managing academic emotions and coping with challenging learning situations, which in turn affects the attainment of learning goals. Second, EI promotes the quality of interactions with peers and teachers, i.e., the social conditions of learning, and can thus contribute to its results. And third, EI—particularly its understanding emotions branch—may be directly involved in mastering contents in the arts and humanities, i.e., in subjects that require emotion knowledge to interpret human actions and artefacts (which are at least partly shaped by emotions). In line with these assumptions, meta-analytic evidence (MacCann et al. 2020) has confirmed that EI is positively associated with academic performance (with an effect size of *ρ* = 0.24), surfacing as its incremental predictor over academic intelligence and personality. Also, in accordance with the proposed mechanisms, the “active ingredients” (MacCann et al. 2020, p. 167) of this predictive effect are EI’s two “strategic branches”—understanding and managing emotions—both of which contribute independently to academic performance.

Beyond cognitive abilities, personality has also been extensively studied as a predictor of academic performance, with most research concentrating on the Big Five (i.e., emotional stability/neuroticism, extraversion, openness, agreeableness, and conscientiousness). Not surprisingly, meta-analytic studies (MacCann et al. 2020; Mammadov 2022; Poropat 2009, 2014) have yielded conscientiousness as the trait with the most robust and strongest, medium-size, effect on the said criterion. Of the Big Five, openness, too, seems to play a significant, albeit smaller, part in students’ performance (Mammadov 2022; Poropat 2009, 2014). As for the remaining three traits, their effects on academic performance tend to be less consistent and—even if statistically significant—usually minor.

While clearly demonstrating that EI predicts academic performance above personality (MacCann et al. 2020), available studies have rarely gone deeper to consider the possible interplay between these predictors. One study that did tackle this issue, testing several plausible predictor arrangements (Jolić Marjanović et al. 2021), produced evidence in favor of a parsimonious model in which conscientiousness, openness, academic (verbal) intelligence, and EI (particularly understanding emotions) each have an independent, direct (positive) effect on academic performance. Nevertheless, the results of this study also allowed for the possibility that the effects of verbal intelligence and openness on student achievement are partially mediated by the understanding emotions branch of EI.

### 1.2. Beyond Individual Achievement: Predicting Students’ Performance in Collaborative Endeavors

The findings presented above concern the prediction of individual academic performance. However, students are increasingly expected to learn and produce work in teams, so that what is assessed as “performance” (projects, presentations, etc.) is in fact the result of a collaborative learning and problem-solving endeavor. Collaborative problem solving (CPS) can be defined as „the joint activity of a dyad or small group whose members are fairly even in terms of competence, responsibility, and power, and thus participate equally in reaching the shared goal of solving a relatively novel and complex task” (Altaras et al. 2025, p. 47; cf. Hesse et al. 2015). Given its importance in today’s “knowledge society”, students’ performance in CPS has been recognized as a new focus of educational assessment and research (Graesser et al. 2018) and has indeed been included as a competence in the Programme for International Student Assessment (PISA; OECD 2017a, 2017b). Naturally, this also calls for empirical insights into the predictors of CPS performance, one question being what role EI and personality play in predicting this criterion.

#### 1.2.1. EI as a Predictor of Performance in CPS

Ever since it was introduced as a new construct, EI has been regarded and studied as a possibly essential resource in teamwork (e.g., Mayer et al. 2000). Focusing on “teamwork” in the academic context—i.e., on collaborative learning and problem solving—it should be noted that the above mechanisms by which EI is expected to impact individual student performance could just as well work to explain students’ performance in CPS. In other words, it is in CPS, too, that EI can affect the management of academic emotions, the social circumstances of learning, and the understanding of certain contents (primarily those from the arts and humanities). As a matter of fact, CPS could provide even more space for the effects of EI to take place, because of the specific socio-cognitive and socio-emotional challenges entailed in collaborating with others. Within Fiore et al.’s (2010a, 2010b) framework of macrocognition in teams, team-level EI could be considered an important dispositional factor supporting the macrocognitive processes of shared situation awareness, model making, and coordination of knowledge across team members.

In line with the above, a recent systematic review (Altaras et al. 2025) has presented mounting empirical evidence of two benefits of higher EI in CPS: First, the team’s EI abilities promote the relational aspects (social processes) of CPS, such as achieving team cohesion, building team trust, and providing team psychological safety; and secondly, team-level EI buffers (moderates) the negative effects of group variables such as team diversity on CPS. In either case, EI ultimately contributes to the performance outcomes of CPS, positively predicting team effectiveness and creativity.

Of note, while the evidence for the positive effects of EI on CPS is rather convincing in terms of the consistency of findings, it still has some considerable gaps: For one, most of it comes from research performed only in tertiary education or in professional contexts. Moreover, extant research has not paid equal attention to all EI branches (focusing more on perceiving and managing than on using and understanding emotions), making it impossible to establish their relative importance in CPS. Additionally, it has not considered the possibility of a “content overlap” between EI and the subject matter of CPS, thus failing to explore the possibility that EI (particularly its strategic branches) can have an additional, direct effect on the quality of the solution produced in CPS, whenever the problem at hand has an “emotional” component to it (i.e., is such that it requires the application of “emotion knowledge”; cf. MacCann et al. 2020).

#### 1.2.2. Personality as a Predictor of Performance in CPS

Regarding personality, the Big Five traits that seem to be most directly related to successful performance in CPS are again conscientiousness and openness. A systematic review of relevant research (Jolić Marjanović et al. 2024) reveals, however, that these traits affect different aspects of CPS and contribute in different (or even inverse) ways: The beneficial effects of higher team-level conscientiousness are largest in long-term CPS and when the task is such that it requires a perseverant and thorough approach from all team members (as in complex written reports or design solutions); team-level openness, on the other hand, has been shown to influence the team’s creative performance, leading to more innovative solutions. Moreover, team openness seems to be particularly important for enhancing team productivity in challenging and uncertain tasks (Bell 2007; Lim et al. 2023).

Apart from being related to CPS performance (in terms of team effectiveness and productivity), team personality can also impact the socio-cognitive, socio-emotional, and other group processes pertinent to CPS. According to the above cited systematic review (Jolić Marjanović et al. 2024), team-level agreeableness is the main and most consistent personality predictor of team processes in CPS, contributing to an open and supportive communication (including the discussion of opposing views), as well as to team cohesion; positive effects on team communication have at times also been reported for team-level openness, extraversion, and emotional stability, while team cohesion has been linked to all of the Big Five traits except openness. Thus, similarly to EI, team personality—primarily team-level agreeableness—can serve to promote the relational aspects of CPS (i.e., team cohesion and communication), which may in turn enhance the quality of the solution produced by the team.

Again—as in the case of EI—the above findings stem primarily from studies conducted with university students or employed adults, still requiring confirmation in the regular school context. Another gap in available research is that it has rarely considered the simultaneous effects of EI and personality on team performance in CPS. More precisely, the few studies that have included both EI and the Big Five as independent variables (Côté et al. 2010; Homan and van Kleef 2022; Paik et al. 2019) have explored their effects on individual performance in teamwork (e.g., on who would emerge as a leader) rather than on the performance of the team as a whole.

### 1.3. The Present Study

The above review has served to demonstrate that EI and personality both play a role in CPS, potentially impacting performance in multiple ways. At the same time, it has revealed some gaps in extant research that we sought to address in the present study. Specifically, our aim was to examine the effects of EI and the Big Five on high-school students’ team performance in CPS, distinguishing between the quality of CPS processes and CPS product, thus allowing us to consider the possibility of both direct and indirect effects of the independent variables on the latter.

Informed by the above reviewed research, we hypothesized that EI abilities would have a positive effect on the quality of team processes in students’ CPS (H1a), thereby also contributing to the quality of the product (H1b), and we expected these effects to be larger for the two strategic EI branches—emotion understanding and emotion management (H1c). Further, considering all three mechanisms by which EI has been proposed to affect academic performance, we assumed that, besides an indirect effect, the two strategic, most knowledge-loaded branches would also have a direct positive effect on the product of CPS (H2).

Similarly, when it comes to personality, we expected that the Big Five would impact CPS processes (H3a) thus having an indirect effect on the CPS product (H3b), while the traits of openness and conscientiousness would also directly contribute to the quality of the solution produced in CPS (H4).

**Figure 1:**
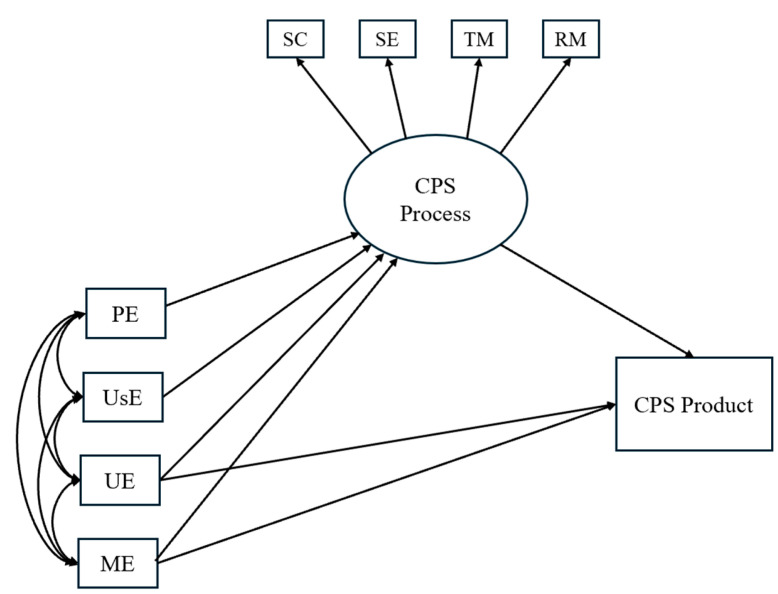
Hypothesized model illustrating the effects of EI branches on CPS processes and product. *Notes.* SC = Socio-Cognitive exchange; SE = Socio-Emotional interaction; TM = Task Management; RM = Relationship Management; PE = Perceiving Emotions; UsE = Using Emotions; UE = Understanding Emotions; ME = Managing Emotions.

**Figure 2:**
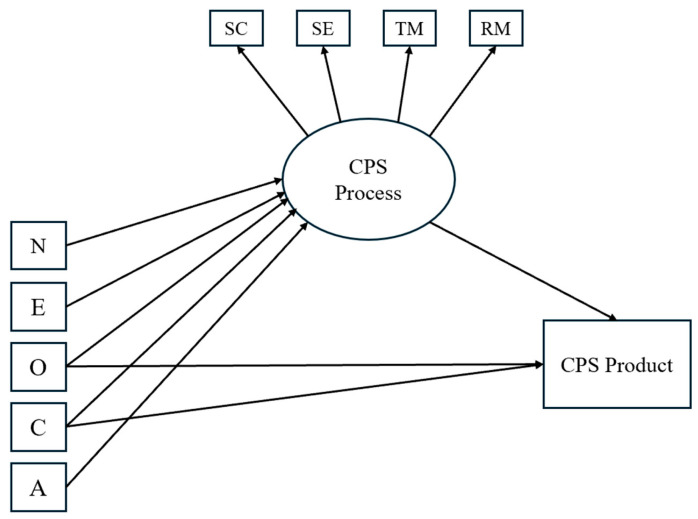
Hypothesized model illustrating the effects of personality traits on CPS processes and product. *Notes.* SC = Socio-cognitive exchange; SE = Socio-emotional interaction; TM = Task Management; RM = Relationship Management; N = Neuroticism; E = Extraversion; O = Openness to Experience; C = Conscientiousness; A = Agreeableness.

**Figure 3:**
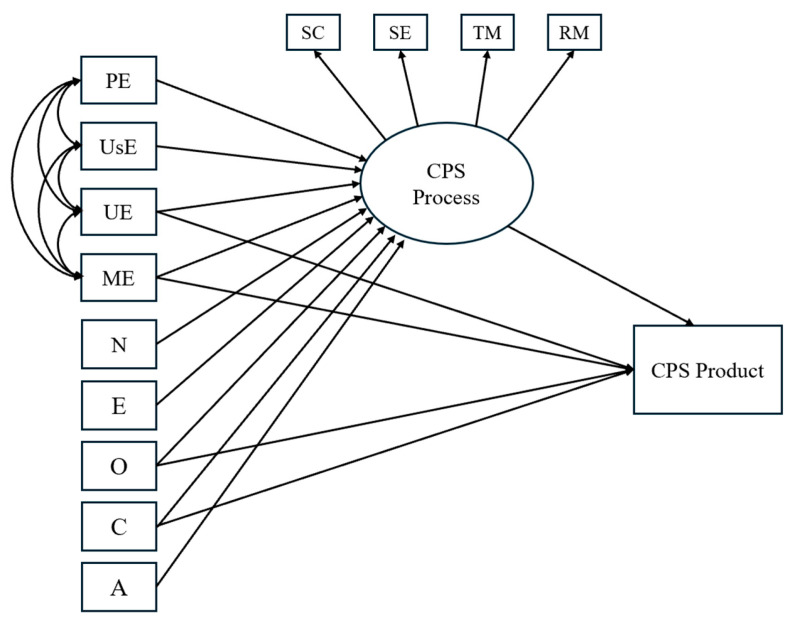
Hypothesized model illustrating the effects of EI branches and personality traits on CPS processes and product. *Notes.* SC = Socio-cognitive exchange; SE = Socio-emotional interaction; TM = Task Management; RM = Relationship Management; PE = Perceiving Emotions; UsE = Using Emotions; UE = Understanding Emotions; ME = Managing Emotions; N = Neuroticism; E = Extraversion; O = Openness to Experience; C = Conscientiousness; A = Agreeableness.

Finally, we hypothesized that EI abilities, on the one hand, and the Big Five personality traits, on the other, would both contribute independently to students’ performance in CPS (H5). A visual representation of the hypotheses is provided in Figure 1, Figure 2 and Figure 3.

## 2. Materials and Methods

### 2.1. Participants

Participants in the study were students from twelve secondary schools (six grammar and six vocational schools) located within a metropolitan area. A total of *N* = 162 tenth-grade students, all aged 16, took part in the study. For the purposes of this study, participants were grouped into gender-homogeneous teams of three, resulting in *n* = 54 triads: 35 (64.8%) female, and 19 (35.2%) male. The distribution was the same for type of school, with 35 triads recruited in grammar schools, and 19 in vocational schools.

### 2.2. Procedure and CPS Task Description

Within each participating school, tenth-grade students were invited to take part in the study through school counsellors (psychologists or education specialists), who also provided a comprehensive explanation of the study’s purpose and procedures to potential participants. Students who agreed to participate were organized into triads, again with the help of school counselors, taking care that all members of a triad come from the same class. Prior to data collection, informed consent was obtained from both the students and their legal guardians, covering participation in the study, as well as the video recording of the CPS sessions. Data collection began with all participants completing an EI test and a personality questionnaire (see Section 2.3).

In the next step, data were collected for student teams (triads), concerning their performance in CPS. Each triad was allotted a 2.5 h time slot to collaboratively solve a complex social problem involving a controversy and pertaining to one of the following domains: ecology, adolescent media use, media content control, or education. More specifically, the problems serving as task material were as follows: (1) How to regulate mineral exploitation to balance between economic and ecological interests; (2) How to regulate adolescent digital media use to balance between teenagers’ need for independence and the potential harms of uncontrolled screen time; (3) How to regulate the broadcasting of reality show programs to balance between media freedom and the potential risk of harmful contents; (4) How to (re)design teaching/learning at school to balance between the transmission of necessary contents and the need to spark students’ interest. The four problem topics had been selected through a pilot study involving ratings of appeal from high-school students and were assigned to the triads randomly.

At the beginning of the CPS session, each triad was given both verbal and written instructions on how to proceed, including the following materials: (1) a detailed description of the problem to be solved and the controversies it involves; (2) a list of criteria requiring that the proposed solution a. consider diverse viewpoints, scientific evidence, and societal interests, b. be agreed-upon by the whole team, c. be well-argued, d. be realistic, and e. be well-elaborated; (3) an answer sheet asking participants to write down a. their final solution, b. the supporting arguments, and c. the sources used in solving the problem. Participants had access to tablets and internet connectivity to gather relevant information throughout the CPS session. Upon completion of the session, each triad submitted their answer sheet as material for the assessment of the CPS product. The entire CPS session was also video recorded as input for the assessment of CPS processes. On average, the CPS sessions lasted 97 min (*SD* = 30; range = 19–167 min).

All testing and CPS sessions were conducted on school premises during regular school hours, with one trained experimenter present. The experimenters were graduate students of psychology or researchers holding a PhD in psychology, all of whom were briefed on how to administer the chosen instruments and supervise the CPS sessions. Specifically, their role was to provide task instructions, monitor progress, and address any technical issues, while refraining from intervening in either test completion or the CPS process.

### 2.3. Measures

#### 2.3.1. Emotional Intelligence 

EI was assessed with the Mayer-Salovey-Caruso Emotional Intelligence Test, version 2.0 (MSCEIT; Mayer et al. 2002), a 141-item performance-based measure yielding four branch scores (i.e., Perceiving Emotions, Using Emotions, Understanding Emotions, and Managing Emotions) and an overall EI score. In solving the test, participants are required either to identify the most appropriate solution to a given problem (multiple-choice format) or to rate the suitability of several possible responses (rate-the-extent format). In this study, the licensed Serbian translation of the test was used; the scoring was performed by the publisher, Multi-Health Systems, using the general consensus method. Previous studies (e.g., Altaras Dimitrijević and Jolić Marjanović 2010; Altaras Dimitrijević et al. 2018a) have provided solid evidence of the structural and convergent-discriminant validity of the Serbian MSCEIT, as well as of its “(psycho)metric equivalence” (Helms 1997) to the US version, revealing response patterns, reliability indices, factor loadings, and correlations with relevant variables (e.g., academic intelligence, personality, trait EI, empathy) comparable to those established for the original test. In the present study, the Spearman–Brown split-half coefficients ranged from 0.72 to 0.88 for branch-level scores, reaching a reliability of 0.92 for the total EI score.

To arrive at a measure of team-level EI, we aggregated team member’s individual EI scores into group means. We opted for this aggregation method not only as the most common way of operationalizing team-level constructs, but also as the one most appropriate given the collaborative nature of the task at hand: When team interaction and performance depend on all team members (rather than on the weakest or the strongest of them), calculating group means has been argued to be the most meaningful aggregation method (Bolin and Neuman 2006; Colquitt et al. 2002).

#### 2.3.2. Personality Traits 

Personality was assessed using the Big Five Inventory (BFI; John et al. 1991), a 44-item questionnaire intended to measure the five broad personality domains: neuroticism (versus emotional stability), extraversion, openness to experience, agreeableness, and conscientiousness. The items consist of brief descriptive statements rated on a 5-point Likert-type scale, ranging from 1 (strongly disagree) to 5 (strongly agree). The BFI has demonstrated strong psychometric properties, which also apply to the Serbian adaptation of the instrument (Smederevac et al. 2024). In the current sample, Cronbach’s alphas ranged from 0.69 to 0.80, indicating adequate internal consistency.

As in the case of EI, individual team members’ scores were aggregated into team personality traits by calculating group means.

#### 2.3.3. CPS Processes 

The quality of CPS processes was rated using a 22-item observational grid pertaining to four categories of group processes established as relevant for productive CPS (Baucal et al. 2023). These are as follows: (1) socio-cognitive exchange (SC), rated with 9 items, including 2 reverse-scored (e.g., Team members sought and/or provided explanations for the presented ideas and suggestions); (2) socio-emotional interaction (SE), rated with 4 items, 1 of which is reverse-scored (e.g., Team members were respectful of each other); (3) task management (TM), rated with 5 items, 1 of which is reverse-scored (e.g., The team planned its approach to solving the task); (4) relationship management (RM), rated with 5 items, including 2 reverse-scored (e.g., Team members worked together to resolve any tensions between them). All items were rated on a 5-point Likert scale, ranging from 0 (not at all) to 4 (to a very large extent). Each CPS session was independently rated by two trained observers. Previous analyses with the current dataset confirmed the grid’s four-factor structure and indicated high inter-rater reliability (ICC = 0.75–0.95), as well as strong internal consistency for the four dimensions (α = 0.79–0.92) (Krstić et al. 2025).

#### 2.3.4. CPS Product 

The quality of the CPS product—i.e., of each triad’s written solution to the problem—was assessed using a structured scoring protocol employing eight criteria: (1) adequacy (0 = not adequate, 1 = adequate); (2) relevance (0 = not relevant, 1 = relevant); (3) realism and elaboration (0 = unrealistic, 1 = realistic but not elaborated, 2 = realistic and elaborated); (4) consideration of multiple perspectives (0 = unilateral solution, 1 = multiple perspectives without integration, 2 = integrated multiple perspectives); (5) quality of argumentation (0 = missing or irrelevant, 1 = poor, 2 = strong argumentation); (6) use of sources (0 = no sources cited, 1 = secondary sources cited, 2 = primary sources cited and specified); (7) creativity (0 = uncreative, 1 = creative and original); (8) overall excellence of the proposed solution (0 = not distinguished by quality, 1 = excellent). Each solution was independently rated by two trained evaluators. The current version of the protocol has demonstrated strong inter-rater reliability (ICC = 0.93) and acceptable internal consistency (α = 0.78) (Krstić et al. 2025).

### 2.4. Data Analyses

First, descriptive statistics, correlations among study variables, and gender differences were analyzed using SPSS version 25 (IBM, Armonk, NY, USA). Next, structural equation modeling (SEM) was performed in AMOS version 21, using the maximum likelihood estimation method, to test the hypothesized direct and indirect effects of (1) EI, (2) the Big Five, and (3) EI and personality combined on both the processes and product of CPS.

Model fit was assessed using several indices with the following thresholds indicating good fit (Hu and Bentler 1999; Kline 2015): (1) a non-significant chi-square statistic (*p* > .05), (2) Comparative Fit Index (CFI) ≥ 0.95, (3) Tucker–Lewis Index (TLI) ≥ 0.95, and (4) Root Mean Square Error of Approximation (RMSEA) < 0.08. In view of the relatively small sample size, to improve the accuracy of standard errors and assess the significance of the indirect effects, a bootstrap estimation procedure with 2000 resamples was employed, using a 95% confidence interval (Nevitt and Hancock 2001). Indirect effects were deemed statistically significant if the confidence interval did not include zero (Hayes 2017).

## 3. Results

### 3.1. Descriptive Statistics and Correlations Between Study Variables

Table 1 presents the basic descriptive statistics for team-level scores and bivariate correlations between all study variables.

Correlations among the four EI branches were moderate to large, consistently positive and statistically significant, as expected. A similar pattern was observed for the four rated CPS processes. All CPS processes were also positively and statistically significantly associated with the quality of the CPS product. EI and the Big Five personality traits were generally unrelated, with the exception of a moderate positive correlation of A with UsE, ME, and total EI.

Regarding the associations between EI and CPS processes, UE, ME, and total EI were statistically significantly positively related to all four CPS processes, with correlations of a moderate size. When it comes to the CPS product, statistically significant correlations—again positive and moderate—were observed with PE, ME, and the total EI score.

Correlations between the Big Five traits and the CPS variables were mostly non-significant, with the following exceptions: N was positively and E was negatively associated with task and relationship management; the latter CPS process was also positively associated with O. The CPS product was positively related to O and negatively to C.

As a first and provisional test of any CPS-task effects, we additionally calculated the correlations between our study variables in a subsample of *n* = 41 triads whose problem topics clearly required emotion knowledge and reasoning (i.e., topics 2–4), excluding the triads who solved the “ecology problem”—the only task that was more scientific in nature (see Section 2.2. Procedure and CPS Task Description). The correlation matrix obtained in this subsample (see Appendix A) revealed a trend towards larger associations between EI and the quality of CPS processes than in the full sample; also, the correlations between O and A, on the one side, and CPS processes and product, on the other, turned statistically significant. Unfortunately, the small size of the subsamples who worked on different problem topics did not permit a more complex analysis of CPS-task effects. In line with this same limitation, all further analyses were conducted on the full sample.

Although we chose group means as the most meaningful method to aggregate team members’ EI and personality scores (see Section 2.3), we also analyzed the correlations between study variables with (a) team standard deviations, (b) team minimum, and (c) team maximum scores on EI and the Big Five as input (see Table A2, Table A3 and Table A4 in Appendix B). Not surprisingly, due to the small size of the teams and consequent restriction in range, the standard deviations in EI abilities or personality did not yield any statistically significant correlations with the CPS variables. Further—in line with previous reports that different aggregation methods tend to be highly correlated and redundant with each other (Barrick et al. 1998)—the team minimum and maximum scores on EI had much the same pattern of correlations with the CPS variables as did the team means: UE and ME were consistently positively associated with CPS processes, and UE also correlated with the quality of the CPS product. For personality, the correlations did differ slightly depending on the aggregation method, with some *r*’s turning statistically significant when looking at the minimum/maximum rather than the mean level of a trait in the team. Nevertheless, considering the nature of our CPS task and the explanation provided above (see Section 2.3 Measures), all further analyses were based on group means as indices of team-level EI and personality.

### 3.2. Validation of the CPS Processes–Product Relationship Model

**Figure 4:**
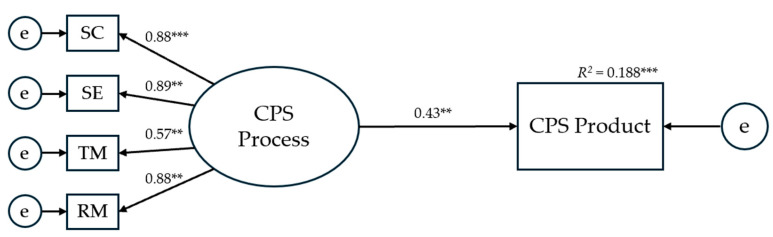
Results for the hypothesized structural equation model testing the direct effect of CPS processes on the CPS product. *Note.* The presented numerical values refer to standardized regression weight coefficients. SC = Socio-cognitive exchange; SE = Socio-emotional interaction; TM = Task Management; RM = Relationship Management; e = error. ** *p* < .01; *** *p* < .001.

Before proceeding to a detailed examination of the effects of the chosen predictor variables (i.e., the four EI branches and the Big Five personality traits) on the assessed aspects of CPS, we first evaluated the validity of a model positing a direct relationship between the four CPS processes and the CPS product. The model demonstrated excellent fit indices (χ^2^ = 5.703, *p* = .336; CFI = 0.995; TLI = 0.990; RMSEA = 0.052), with all hypothesized paths emerging as statistically significant (see Figure 4).

### 3.3. Testing the Direct and Indirect Effects of EI Abilities on CPS Processes and Product

**Figure 5:**
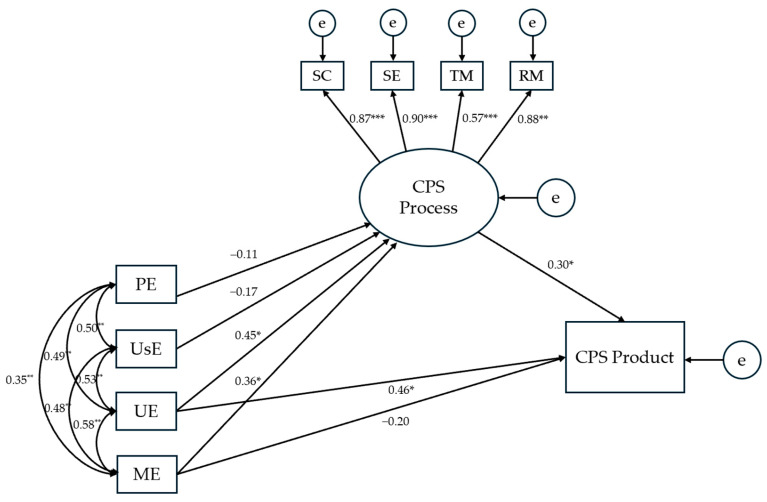
Results for the hypothesized structural equation model testing the effects of EI branches on CPS processes and product. *Notes.* The presented numerical values refer to standardized direct effects coefficients. SC = Socio-Cognitive exchange; SE = Socio-Emotional interaction; TM = Task Management; RM = Relationship Management; PE = Perceiving Emotions; UsE = Using Emotions; UE = Understanding Emotions; ME = Managing Emotions; e = error. * *p* < .05; ** *p* < .01; *** *p* < .001.

The hypothesized model presenting the effects of the four EI branches on CPS accounted for 31.4% of the variance in the CPS product and demonstrated the following model fit indices: χ^2^ = 26.559, *p* = .114; CFI = 0.966; TLI = 0.936; RMSEA = 0.087. Except for the RMSEA value, which slightly exceeds the recommended threshold of 0.08—a deviation that can be justified by the sample size (Kenny et al. 2015)—all other indices indicated a good fit of the model. Standardized regression weights for the direct effects are presented in Figure 5, while those for the indirect effects are summarized in Table 2.

### 3.4. Testing the Direct and Indirect Effects of Personality on CPS Processes and Product

**Figure 6:**
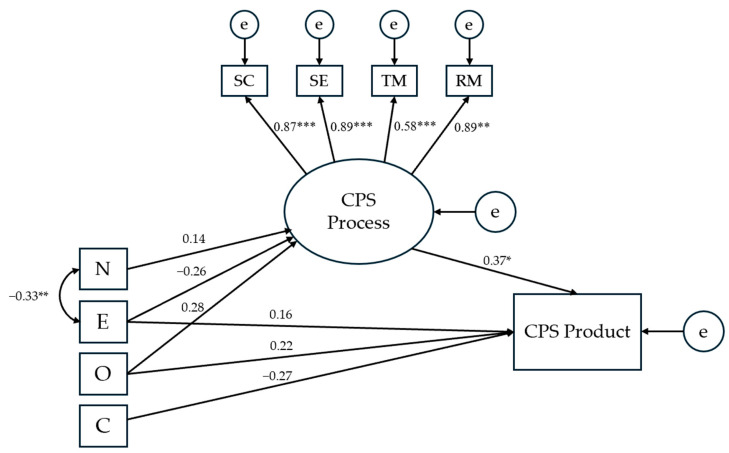
Results for the first modified structural equation model testing the effects of personality traits on CPS processes and product. *Notes.* The presented numerical values refer to standardized direct effects coefficients. SC = Socio-Cognitive exchange; SE = Socio-Emotional interaction; TM = Task Management; RM = Relationship Management; N = Neuroticism; E = Extraversion; O = Openness to Experience; C = Conscientiousness; e = error. * *p* < .05; ** *p* < .01; *** *p* < .001.

The theoretical model depicting the hypothesized effects of the Big Five personality traits on CPS processes and product (Figure 2, Section 1.3) did not have acceptable model fit indices (*χ*^2^ = 48.965, *p* = .036; *CFI* = 0.901; TLI = 0.865; *RMSEA* = 0.096). Consequently, the model was revised based primarily on insights derived from the correlation matrix (Table 1). The revised model, presented in Figure 6, excluded agreeableness and demonstrated good model fit (*χ*^2^ = 32.006, *p* = .127; *CFI* = 0.950; TLI = 0.925; *RMSEA* = 0.079), explaining 28.9% of the variance. However, none of the paths linking personality traits to CPS processes or product were statistically significant.

**Figure 7:**
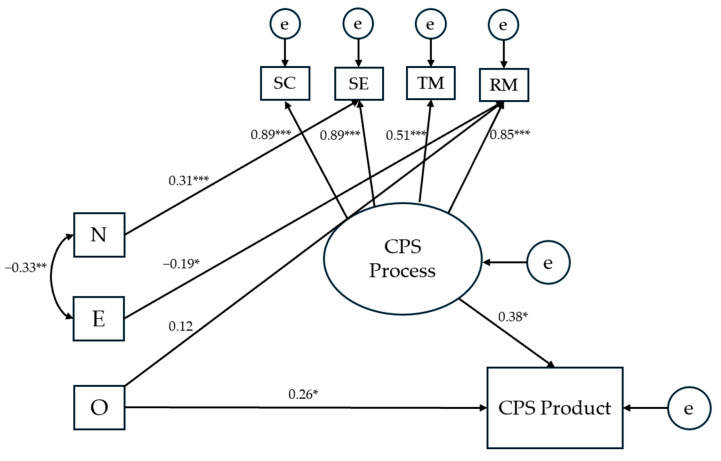
Results for the second modified structural equation model testing the effects of personality traits on CPS processes and product. *Notes.* The presented numerical values refer to standardized direct effects coefficients. SC = Socio-Cognitive exchange; SE = Socio-Emotional interaction; TM = Task Management; RM = Relationship Management; N = Neuroticism; E = Extraversion; O = Openness to Experience; e = error. * *p* < .05; ** *p* < .01; *** *p* < .001.

Modification indices, in conjunction with the correlation matrix, indicated that modelling direct pathways from personality traits to specific CPS processes was more appropriate than modelling a path to the latent CPS processes variable. This second re-specified model (Figure 7) exhibited excellent fit indices (*χ*^2^ = 17.972, *p* = .458; *CFI* = 1; TLI = 1; *RMSEA* = 0) and accounted for 21% of the variance in the CPS product. Detailed estimates for the indirect paths are presented in Table 2.

### 3.5. Testing the Direct and Indirect Effects of EI and Personality on CPS Processes and Product

Consistent with the analytical approach used in previous models, we initially tested our theoretical model positing independent effects of EI branches and personality traits on CPS processes and the product (Figure 3, Section 1.3). However, this model did not produce acceptable fit to the data, as none of the fit indices met the recommended thresholds (*χ*^2^ = 104.147, *p* = .002; *CFI* = 0.853; *TLI* = 0.797; *RMSEA* = 0.104).

**Figure 8:**
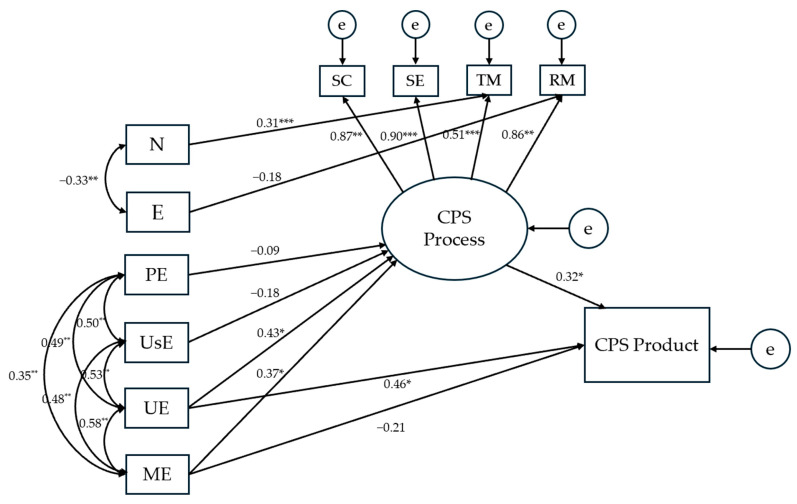
Results for the modified structural equation model testing the effects of EI branches and personality traits on CPS processes and product. *Notes.* The presented numerical values refer to standardized direct effects coefficients. SC = Socio-Cognitive exchange; SE = Socio-Emotional interaction; TM = Task Management; RM = Relationship Management; PE = Perceiving Emotions; UsE = Using Emotions; UE = Understanding Emotions; ME = Managing Emotions; N = Neuroticism; E = Extraversion; e = error. * *p* < .05; ** *p* < .01; *** *p* < .001.

Following the principles applied to revise the model with the Big Five as predictors (see previous section), the initial model was re-specified. A reduced model was retained, incorporating only N and E from the personality set. Structural paths and associations with CPS variables were specified to align with those established in the single predictor-set models. For the EI predictor-set, direct effects were modelled from all EI branches on the latent CPS processes variable and from the two strategic branches to the CPS product. The revised model (Figure 8) accounted for 32.1% of the variance in the CPS product, exhibiting excellent fit indices (*χ*^2^ = 39.442, *p* = .278; *CFI* = 0.981; *TLI* = 0.970; *RMSEA* = 0.049). Standardized regression weights of the direct paths are presented in Figure 8, while indirect effects are given in Table 2.

**Table 2:** Standardized indirect effects and 95% confidence intervals for the final models tested.

Model Tested	Model Pathways	Estimated Effect	95% CI	
			Lower	Upper
EI on CPS performance (Figure 5)	PE → CPS Process → CPS Product	−0.033 (N.S.)	−0.151	0.034
	UsE → CPS Process → CPS Product	−0.050 (N.S.)	−0.224	0.037
	UE → CPS Process → CPS Product	0.136*	0.001	0.448
	ME → CPS Process → CPS Product	0.108*	0.004	0.277
Personality on CPS performance (Figure 6)	N → CPS Process → CPS Product	0.052 (N.S.)	−0.025	0.184
	E → CPS Process → CPS Product	−0.095 (N.S.)	−0.324	0.025
	O → CPS Process → CPS Product	0.103*	0.001	0.297
EI and personality on CPS performance (Figure 8)	PE → CPS Process → CPS Product	−0.029 (N.S.)	−0.147	0.044
	UsE → CPS Process → CPS Product	−0.055 (N.S.)	−0.228	0.040
	UE → CPS Process → CPS Product	0.137 (N.S.)	0.000	0.447
	ME → CPS Process → CPS Product	0.117*	0.011	0.290

*Notes.* PE = Perceiving Emotions; UsE = Using Emotions; UE = Understanding Emotions; ME = Managing Emotions; N = Neuroticism; E = Extraversion; O = Openness to Experience; CPS = Collaborative Problem Solving. * *p* < .05.

## 4. Discussion

In this study, we examined some dispositional predictors of students’ performance in CPS, this criterion being a new focus of educational assessment and research (Graesser et al. 2018). Specifically, we brought together the following two sets of predictors: EI, with its four branches, on the cognitive side, and personality, represented by the Big Five traits, on the noncognitive side. Based on prior research on their role in (1) individual academic performance and (2) CPS outside the school context, we put forth several hypotheses on how these two sets of predictors would affect the processes and the product of students’ CPS. In the following sections, we will revisit these hypotheses and discuss the present findings with regard to extant theory and research.

As indicated by the zero-order correlations (Table 1), the mean level of EI in the student triads was consistently positively associated with CPS processes, including the quality of socio-cognitive exchange and socio-emotional interaction in the triad, as well as the effectiveness of the team’s task and relationship management throughout the CPS session. In accordance with this, the results of SEM supported a model in which EI abilities were assumed to have an effect on (the latent variable of) CPS processes, as stated in H1a (Figure 5). Further, in line with H1b—that by promoting the quality of interaction and organization in CPS, EI would enhance the quality of the solution produced by the team—the model also contained a statistically significant indirect effect on the CPS product. Finally, as assumed in H1c, the established effects were larger (and reached statistical significance) for the two strategic EI branches: Both understanding and managing emotions had a medium-size positive effect on CPS processes (*β* = 0.45 and 0.36), and a small indirect effect on the CPS product (*β* = 0.13 and 0.11). In sum, while not all EI abilities (branches) made an independent contribution to performance in CPS, the overall model, and thus H1, was clearly borne out by the data.

Put into the context of previous research, this result allows us to make several theoretically relevant observations. First of all, the finding that EI contributed to the quality of students’ socio-emotional interaction (SE) and relationship management (RM) in CPS reinforces EI’s previously established role in shaping the relational aspects of CPS (Altaras et al. 2025): In line with what has been reported earlier, the student triads with higher mean levels of the ability to understand and manage emotions tended to be more cohesive, to have a friendlier team atmosphere (SE), and to be better at maintaining equality in teamwork (RM). In addition, the present study showed strategic EI to also promote the socio-cognitive (SC) and task-related (TM) team processes, which pertain to how ideas are exchanged and how the task is approached in CPS. Taken together, these effects of EI on CPS seem to mirror the two indirect mechanisms by which EI has been proposed to affect (individual) academic performance (MacCann et al. 2020): Specifically, the SE and RM loadings on the latent CPS processes variable suggest that EI enhances the quality of the product in part by improving the social/relational circumstances of problem solving and upholding a pleasant working atmosphere; on the other hand, the SC and TM loadings on the same latent variable indicate that EI’s contribution to the quality of the solution is partly achieved through a better understanding and regulation of academic emotions that are likely to arise during an exchange of (opposing) ideas and in attempts to fairly distribute the work. In short, the present findings suggest that both the “building social relationships” and the “regulating academic emotions” mechanism (MacCann et al. 2020) serve well to explain the observed effects of strategic EI on CPS processes and ultimately on the product.

Not only did we find support for the above two indirect paths by which EI abilities have been assumed to impact CPS performance, but our results also revealed a statistically significant direct effect of an EI branch on the quality of the CPS product. Admittedly, H2 was thus only partially supported, as the direct effect held for the understanding but not the managing emotions branch. Although we had reasoned that the latter branch—involving knowledge on emotion regulation strategies—would also be useful in solving such social problems as regulating adolescent media use or balancing the education process (see Section 2), it also makes sense that the direct effect turned out to be fully attributable to understanding emotions as the main seat of emotion knowledge (Mayer and Salovey 1997). The established, medium-size effect (*β* = 0.46) supports the idea of a “content overlap” between the knowledge encompassed by the understanding emotions branch and the knowledge required to solve the kind of social problems presented to participants in this study. As argued in the case of individual academic performance in the language arts and humanities (MacCann et al. 2020, p. 170), a good solution to these problems calls for an understanding of human actions and interactions—and what it is that drives them—and was thus logically impacted by the students’ ability to understand emotions. As further, albeit only tentative, support for the proposition of “content overlap”, the correlations between EI and CPS tended to become larger when we removed the ecology problem from the analyses (see Appendix A), suggesting an increasing effect of EI as the problem to be solved becomes more purely interpersonal/social.

Turning to personality, previous research has shown that all (or rather, any of) Big Five may act to shape CPS processes (particularly team cohesion and communication), which led us to assume that this would be the case in the present study, too. However, contrary to H3, we found only few statistically significant associations between personality and CPS: neuroticism and extraversion were related to both task and relationship management, and the latter variable was additionally associated with openness (Table 1). In line with this, SEM supported a model that includes only two of the Big Five traits (Figure 7), with neuroticism having a positive effect on task management (*β* = 0.31), and extraversion having a negative effect on relationship management (*β* = −0.19).

The first question arising from this finding is how to understand the established effects, given that their direction (positive for neuroticism and negative for extraversion) contradicts previous findings (e.g., van Vianen and De Dreu 2001). As for neuroticism, it is possible that a higher team-level tendency to experience distress and uneasiness can indeed help with task management, because it directs team members towards a more careful planning and monitoring of the problem-solving approach. This would be in line with a qualitative study (Hilliard et al. 2020), in which students acknowledged that anxiety can promote their participation and performance in collaborative learning when they employ problem-focused coping strategies. More broadly, this also resonates with attentional control theory, stating that higher levels of neuroticism may result in comparable or even enhanced levels of performance by exerting compensatory efforts (Eysenck et al. 2007) and reallocating mental resources under demanding task conditions (Smillie et al. 2006). With regard to extraversion, it is our assumption that more extraverted teams are likely to expect group processes “to regulate themselves”, not taking group tensions and conflicts too seriously, thus resulting in a negative impact of this trait on relationship management.

The second question is how to explain the absence of the personality effects that were theoretically expected, but not observed in the present study (e.g., a positive effect of agreeableness on CPS processes). In response to this question, we propose two possible explanations: Laying out the first, psychological one, it is important to note that, in this study, personality was only related to the two “management” aspects of CPS—that is, to the CPS processes (TM and RM) that meant some kind of purposeful intervening in and (re)directing of groupwork (e.g., stopping to make a plan, watch the time, distribute work, or resolve a conflict). In view of this, it is possible that—being from the same class and befriended—the team members were already very much attuned to each other in terms of personality, which hence did not make much of a difference in their socio-cognitive and socio-emotional interaction, up until the point when a challenge in CPS occurred and the triad needed to take ad hoc steps to regulate the problem-solving process. Alternatively, the fact that we observed fewer and smaller than usual effects of the Big Five on CPS could also be a methodological issue. It was often the case in previous research that both personality and the quality of team processes were assessed via self-reports (i.e., with team members assessing, for instance, how cohesive the group is), allowing for the emergence of methodological artefacts (e.g., highly agreeable teams assessing themselves as highly cohesive). In the present study, however, the CPS processes were assessed by an independent observer, precluding such methodological artefacts, and possibly resulting in smaller, yet perhaps more genuine effects of the Big Five on CPS.

When it comes to the direct effects of personality on the CPS product, though, the findings were fully in line with H4: As evident from the correlation matrix (Table 1), both openness and conscientiousness were associated with the quality of the solution produced in CPS—the former trait positively, and the latter one negatively. Accordingly, SEM supported a model in which the paths from both traits to the CPS products were retained, though only the positive effect of openness (*β* = 0.26) was statistically significant (Figure 7). This result is much in line with previous research, when we consider the nature of the task and the criteria for assessing the CPS product employed in our study. Openness has consistently been found to be conducive to creativity (e.g., Feist 2010; Plucker et al. 2010), including creativity in teamwork (Baer et al. 2008), and was understandably helpful in solving a CPS task that was open-ended, new to participants, and which required an original solution (recall that creativity was one of the criteria for rating the CPS product). Conscientiousness, on the other hand, has at times been found to work against (team) creativity and innovation (den Hartog et al. 2020), which also explains its negative association with the CPS product in this study. Overall, our study provides additional evidence on how the effect of conscientiousness can shift from positive to negative depending on the nature of the problem to be solved: While enhancing performance on traditional, convergent scholarly tasks and serving as a major contributor to academic achievement, this trait seems more likely to impede than boost performance in divergent and creative problem solving.

Finally, the findings also supported our assumption that EI and personality would make independent contributions to CPS performance (H5): More specifically, the results of SEM indicated good fit for a model which included the two strategic branches, from the domain of EI, and neuroticism, from the domain of personality, as independent predictors of the criteria in question (Figure 8). Both in this model and in the present results overall, EI abilities had a more prominent effect on CPS processes and product than personality, with the effects of the latter narrowed down to only one trait and one aspect of CPS processes (i.e., task management). The present study thus suggests that the relative contributions of EI and personality may be different when predicting individual student performance vs. performance in CPS: Although strategic EI has already surfaced as the second-best predictor of academic performance—after academic intelligence and before conscientiousness (MacCann et al. 2020)—it seems that EI abilities might explain even more variance relative to personality when the criterion is CPS rather than individual student performance. Again, this would be in line with our premise that collaboration offers a broad playground for understanding and regulating (academic) emotions in self and others, while solving a complex social problem requires much emotion knowledge. As a major theoretical implication for both the field of EI and CPS research, the present findings suggest that EI needs to be considered as an essential team-disposition factor in any comprehensive model proposed to explain students’ performance in CPS (cf. Krstić et al. 2025).

Certainly, the present findings also bear some implications for educational practice. Most obviously, the established positive effects of EI highlight the importance of fostering the respective abilities as an asset to students’ CPS performance. In view of this—and seeking to complement the more general EI training programs on offer (e.g., Brackett et al. 2019)—we have recently proposed a series of classroom-based exercises aimed specifically at enhancing EI in the service of CPS (Jošić and Baucal 2024). With regard to personality traits, the present study supports previous observations that their effects on CPS performance tend to be rather indirect and task-dependent. As already argued elsewhere (Jolić Marjanović et al. forthcoming), we therefore believe that students would profit most from learning when and how a certain trait (e.g., neuroticism or conscientiousness) can work for or against productive CPS.

### Limitations and Directions for Future Research

Considering the methodological issues that might limit the generalizability of the current findings, the first thing to note is the relatively small sample size. Unfortunately, given the duration of the video-recorded CPS sessions and the amount of material to be analyzed, it was impossible to include more student triads in the sample. Similarly, time considerations spoke against a broader assessment of participants’ cognitive abilities. Thus, while letting us gauge the effects of EI and the Big Five relative to each other, the present study could not weigh their effects on CPS relative to academic intelligence. Next, regarding the size and constitution of the teams, it should be observed that all student teams in the present study were made up of three students of the same gender. This leaves the possibility that the effects of EI and personality on CPS processes and overall team dynamics might be different in mixed-gender and larger teams; it is especially the gender-homogeneous composition of the teams that puts constraints on the ecological validity of our findings, as students in Western schools often collaborate in mixed-gender teams. Further, the students in this study were faced with complex, open-ended, social problems and instructed to produce a well-argued, balanced, and creative solution. Changing the nature of the problem to be solved and the criteria by which the product is rated is likely to change the constellation of predictors that affect students’ performance in CPS. Moreover, it should be kept in mind that not all triads were solving the same problem (but one of four possible problems), and this variability of topics is also a factor that could have influenced the present results (as tentatively suggested by our additional analyses, see Appendix A). It should also be observed that we have used the Serbian translations of two instruments developed elsewhere (i.e., the MSCEIT and BFI); despite all psychometric evidence that they are measuring the same constructs as the original forms, the cultural equivalence of tests remains multi-faceted (Helms 1997) and is not assumed here to be absolute. Finally, the small size of the teams in this study made it statistically meaningless to use standard deviations as indices of team-level EI and personality, but the question remains if, in larger teams, within-group variations in EI and the Big Five would have specific effects on CPS, different from those established here for group means.

The latter is precisely one of the issues that could be addressed in future studies. Other directions for future research, derived from the limitations of the present study, would be to explore the effects of EI and personality on CPS a. relative to academic intelligence, b. in teams of students who are not already befriended and are heterogeneous with respect to gender, and c. systematically controlling for the effects of task content. Another meaningful research question is how an EI training would influence the effects of EI on CPS performance: Would there be a “Matthew effect”, with teams initially higher on EI becoming even better in CPS; or would there perhaps be a “compensation effect”, with teams initially lower on EI reaching the same level of CPS performance as high-EI teams after the training? Lastly, given that processing and sharing emotion information in teams is likely to be culture-dependent, it would be useful to examine whether the current findings can be replicated in other cultural settings.

## 5. Conclusions

Having studied the role of EI and the Big Five in predicting high-school students performance in collaboratively solving a complex social problem, requiring a well-argued yet creative solution, we may draw the following conclusions: First, EI made a substantial contribution to students’ performance in CPS, with its strategic branches (understanding and managing emotions) working to enhance the team’s socio-cognitive exchange, social-emotional relations, and task and relationship management, ultimately affecting the quality of the solution produced in CPS. In addition to these indirect effects via CPS processes, the emotion knowledge entailed in the understanding emotions branch contributed directly to the CPS product. Overall, the observed effects support the idea that CPS offers a suitable playground for EI to exert its influence on performance—essentially via the same three mechanisms previously proposed in the context of individual academic achievement—by enhancing the social conditions of problem solving; by facilitating the management of academic emotions during teamwork; and by providing the relevant cognitive input (i.e., emotion knowledge) to solve the (social) problem at hand. Not surprisingly, (strategic) EI emerged as the more prominent predictor of CPS performance than the Big Five. Nevertheless, beyond strategic EI abilities, the personality trait of neuroticism had an independent positive effect on the CPS processes, specifically contributing to the team’s task management (i.e., their planning and monitoring of the problem-solving process). Moreover, with EI “out of the picture”, openness had a direct positive effect on the CPS product, in line with this trait’s well-established role in creative performance. The present results should serve as a useful basis for further studying the effects of EI and personality on high-school students’ CPS, perhaps following the above proposed directions for future research.

## Figures and Tables

**Table 1:** Team-level descriptive statistics and correlations between variables.

Variables	*M (SD)*	1	2	3	4	5	6	7	8	9	10	11	12	13	14
Emotional Intelligence—group mean scores															
1. PE	0.47 (0.63)	1.00	-	-	-	-	-	-	-	-	-	-	-	-	-
2. UsE	0.41 (0.07)	0.50**	1.00	-	-	-	-	-	-	-	-	-	-	-	-
3. UE	0.46 (0.07)	0.49**	0.53**	1.00	-	-	-	-	-	-	-	-	-	-	-
4. ME	0.33 (0.05)	0.35**	0.48**	0.58**	−1.00	-	-	-	-	-	-	-	-	-	-
5. Total	0.42 (0.05)	0.78**	0.75**	0.84**	0.74**	1.00	-	-	-	-	-	-	-	-	-
Big Five personality—group mean scores															
6. N	2.79 (0.56)	−0.02	0.05	0.19	0.18	0.15	1.00	-	-	-	-	-	-	-	-
7. E	3.66 (0.42)	0.06	−0.12	−0.22	−0.07	−0.11	−0.33*	1.00	-	-	-	-	-	-	-
8. O	3.77 (0.43)	0.20	−0.05	0.17	0.19	0.16	0.12	.05	1.00	-	-	-	-	-	-
9. C	3.54 (0.43)	−0.18	0.12	−0.25	−0.10	−0.17	−0.26	.25	−0.06	1.00	-	-	-	-	-
10. A	3.54 (0.36)	0.22	0.31*	0.19	0.30*	0.30*	0.02	−.15	0.15	0.05	1.00	-	-	-	-
Quality of CPS processes															
11. SC	2.47 (0.82)	0.15	0.25	0.51**	0.41**	0.41*	0.14	−0.21	0.18	−0.06	0.16	1.00	-	-	-
12. SE	3.06 (0.78)	0.08	0.13	0.41**	0.49**	0.33*	0.21	−0.19	0.27	−0.22	0.03	0.78**	1.00	-	-
13. TM	2.33 (0.78)	0.23	0.18	0.40**	0.32*	0.37**	0.40**	−0.27*	0.19	−0.05	0.16	0.52**	0.46**	1.00	-
14. RM	2.65 (0.72)	0.15	0.14	0.45**	0.42**	0.36**	0.28*	−0.34*	0.29*	−0.19	0.09	0.77**	0.80**	0.51**	1.00
Quality of CPS product															
15. Total	4.72 (1.89)	0.35*	0.08	0.50**	0.21	0.38**	0.10	.00	0.34*	−0.30*	0.27	0.40**	0.44**	0.31*	0.29*

*Note.* PE = Perceiving Emotions; UsE = Using Emotions; UE = Understanding Emotions; ME = Managing Emotions; N = Neuroticism; E = Extraversion; O = Openness to Experience; C = Conscientiousness; A = Agreeableness; CPS = Collaborative Problem Solving; SC = Socio-Cognitive exchange; SE = Socio-Emotional interaction; TM = Task Management; RM = Relationship Management. * *p* < .05; ** *p* < .01.

## Data Availability

The authors confirm that the data supporting the findings of this study are available within the article. The participants of this study did not give written consent for their data to be shared publicly, and due to the nature of the original data (i.e., video recordings of participants) only the anonymized and scored dataset will be available upon reasonable request.

## Abbreviations

The following abbreviations are used in this manuscript:

EI	Emotional intelligence
CPS	Collaborative problem solving
MSCEIT	Mayer-Salovey-Caruso Emotional Intelligence Test
PE	Perceiving emotions
UsE	Using emotions
UE	Understanding emotions
ME	Managing emotions
SC	Socio-cognitive exchange
SE	Socio-emotional interaction
TM	Task management
RM	Relationship management
PISA	Programme for International Student Assessment
OECD	Organisation for Economic Co-operation and Development
SEM	Structural equation modelling

## Appendix A

**Table A1:** Team-level descriptive statistics and correlations between study variables in the subsample of *n* = 41 excluding the ecology task.

Variables	*M (SD)*	1	2	3	4	5	6	7	8	9	10	11	12	13	14
Emotional Intelligence—group mean scores															
1. PE	0.47 (0.65)	1.00	-	-	-	-	-	-	-	-	-	-	-	-	-
2. UsE	0.42 (0.07)	0.60**	1.00	-	-	-	-	-	-	-	-	-	-	-	-
3. UE	0.46 (0.08)	0.58**	0.56**	1.00	-	-	-	-	-	-	-	-	-	-	-
4. ME	0.33 (0.05)	0.42**	0.53**	0.61**	1.00	-	-	-	-	-	-	-	-	-	-
5. Total	0.42 (0.05)	0.81**	0.78**	0.87**	0.76**	1.00	-	-	-	-	-	-	-	-	-
Big Five personality—group mean scores															
6. N	2.79 (0.54)	−0.03	0.15	0.23	0.37*	0.25	1.00	-	-	-	-	-	-	-	-
7. E	3.65 (0.38)	0.05	−0.09	−0.21	−0.01	−0.10	−0.38*	1.00	-	-	-	-	-	-	-
8. O	3.78 (0.42)	0.21	−0.05	0.16	0.26	0.18	0.03	0.17	1.00	-	-	-	-	-	-
9. C	3.57 (0.42)	−0.23	0.06	−0.25	−0.19	−0.23	−0.25	0.28	−0.06	1.00	-	-	-	-	-
10. A	3.52 (0.37)	0.27	0.29	0.21	0.32*	0.31*	0.10	−0.06	0.23	0.02	1.00	-	-	-	-
Quality of CPS processes															
11. SC	2.61 (0.79)	0.31*	0.33*	0.55**	0.45**	0.50**	0.12	−0.09	0.23	−0.03	0.22	1.00	-	-	-
12. SE	3.24 (0.62)	0.30	0.24	0.48**	0.62**	0.49**	0.30	−0.05	0.46**	−0.15	0.16	0.69**	1.00	-	-
13. TM	2.37 (0.78)	0.27	0.14	0.42**	0.34*	0.39*	0.43**	−0.18	0.15	−0.07	0.23	0.48**	0.48**	1.00	-
14. RM	2.76 (0.68)	0.33*	0.25	0.50**	0.42**	0.47**	0.30	−0.21	0.35*	−0.08	0.17	0.76**	0.76**	0.53**	1.00
Quality of CPS product															
15. Total	4.78 (1.94)	0.38*	0.07	0.51**	0.25	0.40*	0.13	−0.05	0.49*	−0.32*	0.34*	0.33*	0.42**	0.30	0.30

*Note.* PE = Perceiving Emotions; UsE = Using Emotions; UE = Understanding Emotions; ME = Managing Emotions; N = Neuroticism; E = Extraversion; O = Openness to Experience; C = Conscientiousness; A = Agreeableness; CPS = Collaborative Problem Solving; SC = Socio-Cognitive exchange; SE = Socio-Emotional interaction; TM = Task Management; RM = Relationship Management. * *p* < .05; ** *p* < .01.

## Appendix B

**Table A2:** Correlations between study variables, with group standard deviations as indices of team-level EI and the Big Five.

Variables	1	2	3	4	5	6	7	8	9	10	11	12	13	14
Emotional Intelligence—group standard deviations														
1. PE	1.00	-	-	-	-	-	-	-	-	-	-	-	-	-
2. UsE	0.10	1.00	-	-	-	-	-	-	-	-	-	-	-	-
3. UE	0.20	0.17	1.00	-	-	-	-	-	-	-	-	-	-	-
4. ME	−0.14	0.26	0.08	1.00	-	-	-	-	-	-	-	-	-	-
5. Total	0.45**	0.48**	0.48**	0.32*	1.00	-	-	-	-	-	-	-	-	-
Big Five personality—group standard deviations														
6. N	0.07	0.02	0.02	0.01	0.10	1.00	-	-	-	-	-	-	-	-
7. E	0.18	−0.08	0.19	0.08	−0.18	−0.13	1.00	-	-	-	-	-	-	-
8. O	0.16	−0.02	0.01	−0.26	−0.05	0.14	0.14	1.00	-	-	-	-	-	-
9. C	−0.11	0.01	−0.24	0.14	−0.07	0.16	−0.12	−0.25	1.00	-	-	-	-	-
10. A	0.15	0.03	−0.10	−0.05	−0.00	0.01	0.00	−0.07	0.12	1.00	-	-	-	-
Quality of CPS processes														
11. SC	−0.21	−0.05	0.15	−0.06	−0.19	0.03	−0.12	0.03	−0.04	−0.11	1.00	-	-	-
12. SE	−0.11	−0.03	0.14	−0.13	−0.13	0.21	0.01	0.18	0.04	0.02	0.78**	1.00	-	-
13. TM	−0.20	−0.12	−0.03	−0.04	−0.24	0.03	−0.09	0.07	−0.07	0.07	0.52**	0.46**	1.00	-
14. RM	−0.19	−0.05	0.12	−0.06	−0.15	0.14	−0.08	0.08	0.07	−0.04	0.77**	0.80**	0.51**	1.00
Quality of CPS product														
15. Total	0.02	0.10	0.24	−0.05	−0.08	−0.16	−0.18	−0.12	−0.05	0.05	0.40**	0.44**	0.31*	0.29*

*Note.* PE = Perceiving Emotions; UsE = Using Emotions; UE = Understanding Emotions; ME = Managing Emotions; N = Neuroticism; E = Extraversion; O = Openness to Experience; C = Conscientiousness; A = Agreeableness; CPS = Collaborative Problem Solving; SC = Socio-Cognitive exchange; SE = Socio-Emotional interaction; TM = Task Management; RM = Relationship Management. * *p* < .05; ** *p* < .01.

**Table A3:** Correlations between study variables, with group minimum scores as indices of team-level EI and the Big Five.

Variables	1	2	3	4	5	6	7	8	9	10	11	12	13	14
Emotional Intelligence—group minimum scores														
1. PE	1.00	-	-	-	-	-	-	-	-	-	-	-	-	-
2. UsE	0.38**	1.00	-	-	-	-	-	-	-	-	-	-	-	-
3. UE	0.40**	0.41**	1.00	-	-	-	-	-	-	-	-	-	-	-
4. ME	0.14	0.43**	0.51**	1.00	-	-	-	-	-	-	-	-	-	-
5. Total	0.69**	0.69**	0.80**	0.60**	1.00	-	-	-	-	-	-	-	-	-
Big Five personality—group minimum scores														
6. N	−0.07	0.18	0.17	0.05	0.13	1.00	-	-	-	-	-	-	-	-
7. E	−0.02	−0.02	−0.14	−0.05	−0.15	−0.37**	1.00	-	-	-	-	-	-	-
8. O	0.20	−0.00	0.16	0.13	0.08	0.09	−0.10	1.00	-	-	-	-	-	-
9. C	−0.22	0.04	−0.22	−0.03	−0.13	−0.26	0.18	−0.24	1.00	-	-	-	-	-
10. A	0.16	0.38**	0.13	0.17	0.29*	−0.05	−0.10	0.04	0.21	1.00	-	-	-	-
Quality of CPS processes														
11. SC	0.16	0.20	0.40**	0.40**	0.42**	0.17	−0.20	0.09	−0.01	0.20	1.00	-	-	-
12. SE	0.09	0.08	0.29**	0.47**	0.32*	0.14	−0.26	0.10	−0.17	0.03	0.78**	1.00	-	-
13. TM	0.21	0.17	0.33**	0.25	0.35**	0.38**	−0.35**	0.09	−0.05	0.09	0.52**	0.46**	1.00	-
14. RM	0.17	0.10	0.35**	0.38**	0.35**	0.27*	−0.36**	0.16	−0.14	0.08	0.77**	0.80**	0.51**	1.00
Quality of CPS product														
15. Total	0.24	−0.02	0.27*	0.22	0.30*	0.17	−0.08	0.33	−0.18	0.19	0.40**	0.44**	0.31*	0.29*

*Note.* PE = Perceiving Emotions; UsE = Using Emotions; UE = Understanding Emotions; ME = Managing Emotions; N = Neuroticism; E = Extraversion; O = Openness to Experience; C = Conscientiousness; A = Agreeableness; CPS = Collaborative Problem Solving; SC = Socio-Cognitive exchange; SE = Socio-Emotional interaction; TM = Task Management; RM = Relationship Management. * *p* < .05; ** *p* < .01.

**Table A4:** Correlations between study variables, with group maximum scores as indices of team-level EI and the Big Five.

Variables	1	2	3	4	5	6	7	8	9	10	11	12	13	14
Emotional Intelligence—group maximum scores														
1. PE	1.00	-	-	-	-	-	-	-	-	-	-	-	-	-
2. UsE	0.43**	1.00	-	-	-	-	-	-	-	-	-	-	-	-
3. UE	0.31*	0.45**	1.00	-	-	-	-	-	-	-	-	-	-	-
4. ME	0.34*	0.48**	0.45**	1.00	-	-	-	-	-	-	-	-	-	-
5. Total	0.57**	0.68**	0.77**	0.71**	1.00	-	-	-	-	-	-	-	-	-
Big Five personality—group maximum scores														
6. N	−0.03	0.02	0.16	0.24	0.06	1.00	-	-	-	-	-	-	-	-
7. E	0.14	−0.16	−0.13	−0.05	−0.05	−0.15	1.00	-	-	-	-	-	-	-
8. O	0.10	−0.13	0.19	0.10	0.14	0.11	0.21	1.00	-	-	-	-	-	-
9. C	−0.16	−0.04	−0.25	0.12	−0.21	−0.06	0.20	0.07	1.00	-	-	-	-	-
10. A	0.19	0.03	0.22	0.27**	0.13	0.16	−0.05	0.14	−0.01	1.00	-	-	-	-
Quality of CPS processes														
11. SC	−0.02	0.22	0.53**	0.37**	0.34*	0.15	−0.05	0.17	−0.07	0.14	1.00	-	-	-
12. SE	−0.01	0.07	0.40**	0.37**	0.29*	0.27	−0.00	0.31**	0.16	0.06	0.78**	1.00	-	-
13. TM	0.08	0.17	0.34**	0.24	0.20	0.34*	−0.09	0.22	0.11	0.22	0.52**	0.46**	1.00	-
14. RM	−0.02	0.10	0.44**	0.35**	0.32*	0.31	−0.16	0.29	0.11	0.08	0.77**	0.80**	0.54**	1.00
Quality of CPS product														
15. Total	0.40**	0.09	0.47**	0.17	0.29*	0.01	0.08	0.28*	−0.31*	0.32*	0.40**	0.44**	0.31*	0.29*

*Note.* PE = Perceiving Emotions; UsE = Using Emotions; UE = Understanding Emotions; ME = Managing Emotions; N = Neuroticism; E = Extraversion; O = Openness to Experience; C = Conscientiousness; A = Agreeableness; CPS = Collaborative Problem Solving; SC = Socio-Cognitive exchange; SE = Socio-Emotional interaction; TM = Task Management; RM = Relationship Management. * *p* < .05; ** *p* < .01.
